# Burnout and patient safety: navigating between exaggerated evidence and warranted assertability

**DOI:** 10.3389/fpsyg.2026.1801061

**Published:** 2026-04-27

**Authors:** Anthony Montgomery, Vilma Chalili, Ilias Maliousis, Olga Lainidi

**Affiliations:** 1 School of Psychology, Northumbria University of Newcastle, Newcastle upon Tyne, United Kingdom; 2 School of Medicine, Aristotle University of Thessaloniki, Thessaloniki, Greece; 3 Greek Association of Alzheimer’s Disease and Related Disorders, Thessaloniki, Greece; 4 Department of Psychology, University of Essex, Colchester, United Kingdom; 5 School of Psychology, University of Leeds, Leeds, United Kingdom

**Keywords:** healthcare, job burnout, measurement, patient safety, sustainability

## Abstract

Job burnout is widely assumed to be a key driver of patient safety failures in healthcare, underpinning numerous research programmes and policy interventions. However, the strength and quality of the empirical evidence supporting this assumption remain unclear. Using a critical interpretive synthesis, we reviewed quantitative systematic reviews and interrogated the primary studies they included, allowing direct comparison between review-level conclusions and the underlying empirical evidence. Across eight reviews, only a minority of primary studies examined objective safety outcomes, and findings were inconsistent. Despite this, review conclusions often implied stronger and more generalisable effects than the evidence warranted. We identify four recurring problems in the literature: a narrow occupational focus, limited theoretical positioning of burnout within patient safety systems, extrapolation beyond objective evidence, and conflation of reported with observed safety events. Drawing on the concept of warranted assertability, we argue that burnout cannot currently be justified as a direct predictor of patient safety outcomes. Instead, burnout is better understood as a system-level condition that shapes care processes, reporting practices, and organisational adaptation. We conclude by proposing an open-systems framework for theorising burnout and patient safety that aligns psychological constructs with the realities of complex socio-technical healthcare systems.

## 1 Introduction

The ways in which institutional processes, clinical environments, and the behaviour of healthcare staff influence patient safety have been investigated by scholars across a range of disciplines, including medicine, health sciences, and psychology (Vincent et al., 1998; Sattar et al., 2024). There is a reasonable assumption that the processes of patient safety delivered in healthcare systems are shaped by or “rooted in” psychological phenomena such as job burnout, psychological safety, team building, safety culture, effective leadership, and knowledge sharing (and many more) (Edmondson, 1999; Vincent et al., 2014). Within this body of work, job burnout has emerged as one of the most frequently invoked psychological explanations for patient safety failures, based on the assumption that emotionally exhausted and cognitively depleted staff are more likely to make errors, disengage from safe practice, or fail to detect emerging risks. Burnout is now routinely embedded in patient safety frameworks and policy discussions, often treated as a key determinant of safety performance alongside technical and procedural safeguards (Berhanu et al., 2026).

Despite the decades of patient safety efforts, preventable harm remains a common occurrence across clinical settings (Newman-Toker et al., 2021). For instance, diagnostic errors in the United States result in death or serious harm for nearly 800,000 people each year, and adverse events affect approximately one in four medical hospitalisations (Newman-Toker et al., 2023). The failure to reduce preventable, familiar harms suggests that the systems remain vulnerable to multiple kinds of failures, including those that emerge in less predictable ways (Vincent et al., 2014). Procedural and technical aspects alone have not been sufficient. A great emphasis is placed on psychological and organisational determinants contributing to safe care, with psychologically driven interventions aiming to improve clinical work (Dixon-Woods et al., 2012). Consequently, a substantial research and intervention literature has developed in which burnout (and related organisational variables) is frequently positioned as a modifiable risk factor for adverse events, medical error, and reduced quality of care (e.g., Garcia et al., 2019; Moffatt-Bruce et al., 2019).

However, the evidence is equivocal. For example, Hoff et al. (2004), in an intensive review of clinical and health services literature, found little evidence for asserting the importance of any individual, group, or structural variable in error prevention or enhanced patient safety. Their interpretation can be read as implying that care may rest fundamentally on *lege artis* standards, medical procedures, sufficient resources, and ongoing professional training rather than on psychological constructs per se. Although this critique pre-dates much contemporary burnout and organizational research, its central concern remains unresolved: the tendency to infer causal importance from psychological correlates without sufficient empirical scrutiny and questioning of what the evidence means. Fundamental burnout-related constructs, including job demands and resources, workload, autonomy and resource depletion are widely framed as integral parts of healthcare’s sociotechnical systems (Carayon et al., 2013). This paper builds upon complexity theories such as socio-technical theory, which was a by-product of a study by the UK Tavistock Institute on the coal mining machinery (van Eijnatten, 2013; Trist, 1981). Socio-technical theory has at its core the idea that the design and performance of any organisational system can only be understood and improved if both ‘social’ and ‘technical’ aspects are brought together and treated as interdependent parts of a complex system. The mechanisms of burnout are unlikely to exert direct and linear effects on clinical outcomes; instead, they shape care processes and the system’s adaptability, with implications for the probability and/or severity of harm (Hollnagel, 2014).

In this paper, we revisit and extend Hoff et al.’s (2004) critique by focusing specifically on job burnout and its relationship with objective patient safety outcomes. Our aim is not to assess whether job burnout “matters” for patient safety, but to clarify how it matters, under what conditions, and with what evidential support burnout can be argued to influence patient safety and outcomes. Accordingly, this paper has three objectives: first, to synthesise the accumulated evidence from existing quantitative systematic reviews examining associations between job burnout and objective patient safety outcomes, with close interrogation of the primary studies they include; second, to critically analyse the methodological, theoretical, and conceptual assumptions underlying claims about burnout–patient safety relationships; third, to identify the risks associated with inferring causal or linear effects from the available research evidence. To achieve these aims, we employed a critical interpretive synthesis (CIS) (Dixon-Woods et al., 2006) of quantitative systematic reviews and meta-analyses reporting associations between burnout and objective patient safety outcomes. We draw on the concept of warranted assertability (Dewey, 1939) to distinguish between propositions that are well supported and those that remain speculative or weakly justified, in order to evaluate whether commonly cited claims about burnout and patient safety are proportionate to the strength, consistency, and scope of the available evidence. We conclude the synthesis by outlining implications for research, practice and theory.

### 1.1 Operationalising patient safety

We begin by clarifying how patient safety has been defined and operationalised in relevant research. Although definitions of patient safety rest on the principle of “first do no harm,” they are operationalised in different ways depending on the outcome (Rodziewicz et al., 2024) and have been conceptualised in two principal ways. Safety I defines safety as the absence of adverse events and focuses on analysing incidents in which harm occurred, asking what went wrong, and how it might have been prevented. Safety II conceptualises safety as the presence of successful performance under varying conditions and emphasises reducing risk, focusing on effective and high-quality clinical work and asking how harm is avoided and how systems adapt (Hollnagel et al., 2015). More broadly, patient safety can be understood as a coordinated set of activities shaping cultures, processes, procedures, behaviours, technologies and environments in ways that sustainably reduce risks, as well as minimise avoidable harm and its impact (WHO, 2023). This framing positions patient safety as a dynamic and more holistic capability across individual, team, and organisational levels to detect, prevent, and mitigate avoidable harm. In such a framework, psychological processes (e.g., job burnout) and functions can be positioned as “peripheral systems” informing the “central system” (i.e., patient safety outcomes) through interrelated pathways.

In this paper, we distinguish between objective and subjective patient safety outcomes. Objective indicators refer to formally recorded clinical events, including adherence or deviation from protocols, adverse events, complication rates, mortality, misdiagnoses, and reporting frequencies. Subjective indicators, by contrast capture perceptions of safety grounded in culture, climate, and experiences, typically assessed through surveys and narratives.

Objective patient safety outcomes are central to regulatory, legal, and policy decision-making and are frequently treated as the ultimate criteria by which the safety of healthcare systems is judged. For this reason, our focus on objective patient safety outcomes is analytical rather than normative: we examine these outcomes precisely because they are rhetorically privileged in policy and practice as the most robust indicators of safety. At the same time, we explicitly recognise that a lot of the objective safety outcomes are not behaviourally neutral. Their occurrence, detection, classification, and reporting are shaped by individual and collective behaviour, professional norms, organisational culture, and structural incentives. Consistent with multilevel models of safety (Vu and De Cieri, 2015), objective indicators often reflect not only underlying safety performance but also psychologically mediated processes such as reporting behaviour, risk appraisal, communication, and trust. Thus, we use the term ‘objective’ in this administrative sense to denote outcomes that are formally recorded within healthcare systems, rather than to imply that they are independent of human behaviour, judgement, or organisational context. This perspective positions objective safety indicators as system-level outcomes that are partially co-produced by psychological and organisational conditions, rather than isolated technical endpoints.

However, objective harm indicators and adverse events reporting represent only one aspect of safety evaluation, failing to capture some forms of harm (e.g., routine violations) (Vincent et al., 2014) and relying on organisational priorities and reporting behaviour. Behaviours and beliefs concerning burnout are embedded within local norms shaped by both individual and unit-level psychological characteristics such as culture, trust, fear of blame, perceived efficacy of reporting, ethical behaviour, and professional identity. Crucially, organisational research on goal setting further suggests that highly salient performance targets, including “the illumination of harm” (i.e., objective harm outcomes), can unintentionally increase the risk of dysfunctional or unethical responses (e.g., avoiding reporting of harm when it is not serious), especially when target attainment becomes the valued measure (Ordóñez and Welsh, 2015). For these reasons, subjective and organisational indicators are best understood as complementary to objective harm metrics, helping to distinguish safer care from reduced/biased perception. This reframes policy questions from “how many more skills do clinicians need?” to “what must organisations provide so that safe care is the default?” For example, regulatory reporting systems can illuminate how organisational practices shape the detection of an adverse event. In the UK, the Yellow Card scheme supports pharmacovigilance, and the Yellow Card Hospital approach in Wales has been reported to be associated with significant increases in reporting (app. 81%) following targeted training and awareness initiatives. Persistent barriers, including limited awareness and uncertainty about what constitutes a reportable adverse drug reaction, indicate that “safety data” are partly co-produced by system and measurement designs and modifiable psychological determinants, such as confidence and perceived efficacy of reporting behaviour (Pharmaceutical Journal, 2025). As a result, even when an adverse event has occurred, its classification, particularly in terms of attributed severity and clinical significance may vary in practice, shaping what is ultimately recorded as an “objective” patient safety outcome.

Against this background, the present paper focuses on objective patient safety outcomes not because they provide a complete or behaviourally neutral representation of safety, but because they are most frequently invoked as the most “robust” evidential basis for claims that job burnout compromises patient safety. Subjective patient safety assessments, such as safety culture and safety climate surveys, play an important role in understanding perceptions and experiences of safety; however they are typically derived from self-report data collected from the same respondents as psychological predictors, increasing susceptibility to response artefacts that can inflate associations and yield overly consistent findings (Podsakoff et al., 2003). In addition, these measures raise concerns about ecological fallacy (Glick and Roberts, 1984; Jackson et al., 2006) and the validity of inferences drawn about system-level safety performance (O’Connor et al., 2011), with often limited predictive validity for objective safety outcomes (Vu and De Cieri, 2015); they reflect how safe care feels, not whether harm was detected, or was prevented. As such, subjective indicators are less appropriate for evaluating claims about the downstream consequences of burnout for reported patient harm.

### 1.2 Why we expect burnout and patient safety outcomes to be connected?

Burnout, characterized by emotional exhaustion, depersonalization, and decreased personal accomplishment is a significant challenge among healthcare professionals (Maslach and Leiter, 2017), and has the potential to signal risks for patients and colleagues through higher rates of absenteeism, presenteeism, turnover, and medical error (Kieft et al., 2014; Hall et al., 2016). Burnout may also be implicated in a reciprocal process, whereby involvement in safety incidents and errors may generate moral residue that accumulates over time, undermining healthcare workers’ mental well-being and performance. Previous research has established healthcare workers as secondary victims of medical error (Wu, 2000; Ozeke et al., 2019), with the identification of a range of emotional and psychological repercussions (Wu, 2000; Waterman et al., 2007). The phenomenon of the second victim has been defined as “any health care worker, directly or indirectly involved in an unanticipated adverse patient event, unintentional healthcare error, or patient injury, and who becomes victimized in the sense that they are also negatively impacted” (Vanhaecht et al., 2022). The psychological toll can lead to decreased attention, increased anxiety and fear, which impair the ability of healthcare professionals to deliver safe, effective care. Thus, negative outcomes regarding patient safety may both contribute to burnout and, in turn, be exacerbated when burnout increases. For example, the WHO and the Institute of Medicine (IOM) suggest that there are six dimensions that impact the quality of healthcare: effectiveness, efficiency, accessibility, equitability, acceptability and safety (Institute of Medicine, 2001; WHO, 2006). Burnout can signal problems with all six, meaning that when and where well-being is compromised, patient interactions may also be negatively affected – and vice versa (Montgomery et al., 2021). There is evidence that higher levels of healthcare workers’ burnout are consistently linked to poorer perceived patient safety and reported quality of care (Tawfik et al., 2018; Van Gerven et al., 2016).

Maslach (2018, p.11) notes that “When workers are experiencing burnout, they are overwhelmed, unable to cope, unmotivated, and display negative attitudes and poor performance. They have lost any passion for the work they do, and do not take pride in what they might accomplish. Rather than trying to do their very best, they do the bare minimum (i.e., just enough to get by and still get paid)”. This description highlights how burnout can be understood as an adaptive response to chronically demanding work environments, characterised as ‘burnout shops’ by Maslach (2018). Safety in any industry is dependent on high-reliability organisations, and the many examples of poor care in healthcare (e.g., the Mid Staffordshire scandal, Bristol Heart scandal) illustrate the risks that arise when workforce energy levels and resilience are depleted. The Mid Staffordshire scandal (2005–2009) involved widespread failings in basic patient care within an NHS hospital trust in England, leading to avoidable patient harm and deaths, and prompted a major public inquiry into organisational culture, leadership, and system oversight (Francis, 2013). The Bristol Royal Infirmary inquiry (1990s) concerned elevated mortality rates in paediatric cardiac surgery, revealing systemic governance and monitoring failures within the NHS (Kennedy, 2001). Congruently, there is growing evidence that cognitive functioning (e.g., attention, executive control, decision-making) is directly related to burnout (Deligkaris et al., 2014).

Ultimately, the link between burnout and patient safety is based on the way its effects reverberate both inward and outward for healthcare workers. At the individual level, burnout is linked with poor mental health, including depression, substance use and abuse, and suicidal tendencies (West et al., 2018). At the system level, burnout is associated with decreased organizational productivity, increased errors, and lower patient satisfaction (Dewa et al., 2017). The costs of burnout, such as productivity losses as well as turnover cost increments, are significant (e.g., estimated to be $4.6 billion annually in the United States, Han et al., 2019). Thus, the connection between burnout and patient safety is unsurprising, but our understanding of how, why and when burnout increases the probability of harm is incomplete. Rather than positioning burnout solely as a driver of unsafe care, these patterns suggest that burnout may also serve as an indicator – or even a consequence - of chronically unsafe or unsupportive systems in which patient safety has already been compromised. Therefore, a CIS approach is warranted to allow us to question, reformulate and reconceptualise the connection.

## 2 Critical interpretative synthesis of the evidence on job burnout and objective patient safety outcome

The Critical Interpretative Synthesis (CIS) is a literature review approach that is aligned with our aim of critically examining how organisational psychological variables are conceptualised and enacted in relation to incident reporting, and patient concerns systems. As Dixon-Woods et al. (2006, p. 10) note: “CIS does not aim to offer a series of pre-specified procedures for the conduct of review”. It explicitly acknowledges the “authorial voice”. A core feature of CIS is the production of ‘synthesising arguments’ (Dixon-Woods et al., 2006). The synthesis was guided by the following compass question: “What is the evidence supporting the role of job burnout in explaining and understanding patient safety?”. We focused our synthesis on quantitative systematic reviews and/or meta-analyses published up to December 2025 that examined associations between job burnout and objectively recorded patient safety outcomes. Building on this synthesis, we then applied the lens of warranted assertability (Dewey, 1939) to evaluate the degree to which different elements of job burnout can legitimately be said to contribute negatively to objective patient safety outcomes. Warranted assertability was used to assess whether review-level claims were proportionate to the strength, consistency, and scope of the underlying evidence.

In terms of the scope of the synthesis, we did not restrict inclusion to any particular disciplinary field (e.g., medicine, nursing, psychology, management, health services research, or information systems). Reviews were eligible irrespective of their disciplinary origin, provided they quantitatively examined associations between job burnout and objectively recorded patient safety outcomes. At the same time, this study did not employ a protocol-driven systematic search strategy of the kind required in PRISMA-style systematic reviews. Consistent with the principles of Critical Interpretive Synthesis (Dixon-Woods et al., 2006), searching and selection were iterative and guided by conceptual relevance and empirical sufficiency rather than exhaustive coverage. We therefore deliberately bounded the synthesis to (a) the construct of job burnout as defined in the review literature, and (b) outcomes that were formally recorded within healthcare systems, in order to directly interrogate the evidential basis for claims that burnout predicts objective patient outcomes. Reviews were eligible for inclusion if they met all of the following criteria: (1) were published in peer-reviewed journals up to December 2025 with no initial time limits set; (2) identified themselves as a quantitative systematic review and/or meta-analysis; (3) explicitly examined job burnout as a defined construct (e.g., emotional exhaustion, depersonalisation/cynicism, reduced personal accomplishment, or validated composite burnout measures); (4) stated that they assessed patient safety using objectively recorded or administratively documented indicators (e.g., mortality, healthcare-associated infections, adverse events, chart-audited medical errors, complication rates, failure-to-rescue, length of stay); and (5) reported empirical associations between burnout and these outcomes. Table 1 provides a detailed description of the reviews analysed. Thirty-seven reviews were initially identified, of which eight met inclusion criteria and formed the basis of the synthesis. Twenty-nine were excluded because they did not include studies directly assessing job burnout or were narrative or literature reviews. The eight included reviews were examined in detail, alongside close reading of the individual primary studies they included, to determine (a) whether patient safety outcomes were operationalised using objective indicators rather than survey-based measures, (b) the specific nature and direction of the reported relationships, and (c) how these findings were reflected in, or diverged from, the overarching conclusions drawn by the review authors. Given the absence of a consistently agreed definition of ‘objective’ patient safety outcomes and the heterogeneity of indicators across reviews, we adopted an intentionally inclusive operationalisation for the purposes of synthesis. This two-stage, review-within-review approach allowed a critical comparison between primary evidence and higher-level syntheses, supporting a more nuanced interpretation of how job burnout relates to objectively recorded patient safety outcomes.

**Table 1:** Quantitative reviews assessing the relationship between job burnout and objective measures of patient safety.

Review no.	**Study**	**Review type** **(No of studies:no of studies assessing objective measures)**	**Psychological variables**	**Main result claimed by authors**	**Actual evidence**
1	Jun et al. (2021)	Systematic Review (20:2)	Burnout	Nurse burnout is associated with worsening safety and quality of care,	Only two studies found a relationship: 1: Emotional Exhaustion associated with standardised mortality ratios, burnout not associated with length of stay^1^ 2: Burnout associated with increased rates of urinary tract infection and surgical site infections^2^
2	Garcia et al. (2019)	Systematic Review and Meta-Analysis (19:2)	Burnout	There is a relationship between high levels of burnout and worsening patient safety.	Only two studies had findings related to objective patient outcomes: 1: Burnout rates showed no correlation with healthcare-associated infection^3^ 2: Burnout was not associated with medical errors^4^
3	Dewa et al. (2017)	Systematic Review (12:1)	Burnout	Moderate evidence for the association between burnout and safety aspects of healthcare	Only one study assessed errors based on chart audits: 1: No relationship between burnout and medical errors^5^
4	Hall et al. (2016)	Systematic Review (46:7)	Burnout, Well-being	The majority of studies provided evidence that both wellbeing and burnout are associated with patient safety.	Only 7 Studies assessed objective measures: 1: correlation between average sleep hours and medication errors^6^ 2: no associations between chart audit errors and wellbeing or burnout^7^ 3: Medication events were associated with workload and higher emotional stress scores^8^ 4: stress scores correlated with patient incidents^9^ 5: burnt-out residents made more errors than non-burnt-out residents when using subjective self-reported, but not objective, measures of error^10^ 6: Depression was an independent risk factor for error (as assessed by chart audit), but not burnout^11^ 7: association between patient-to-nurse ratio and urinary tract infection and surgical site infection^12^
5	Li et al. (2024)	Systematic Review and Meta-Analysis (85:4)	Nurses’ Burnout	Nurse burnout was found to be associated with lower health care quality and safety	1: In one study, EE was associated with rates of pneumonia and pressure ulcers. Reduced PA was associated with rates of urinary catheter^13^ 2: In one study, burnout was associated with 30-day hospital mortality, and Failure to Rescue^14^ 3: In one study, no association between mortality rate and burnout^15^ 4: In one study, no association between adverse events and burnout^16^
6	Al-Ghunaim et al. (2022)	Systematic Review and Meta-Analysis (9:0)	Surgeon Burnout	An association between higher levels of emotional exhaustion and a greater likelihood of being involved in a patient safety incident	All studies were self-reports. EE, but not DP associated with patient safety incident reporting.
7	Welp and Manser (2016)	Systematic review 25:6 (Wellbeing)	Teamwork Burnout Other well-being-related variables	Limited and mixed support for the associations between well-being, teamwork and safety	Mixed and contradictory findings concerning teamwork and well-being Teamwork & Patient Safety 1: Burnout associated with increased rates of urinary tract infection and surgical site infections^17^ 2: Burnt-out residents made more errors than non-burnt-out residents when using subjective self-reported, but not objective, measures of error^18^ 3: Burnout was not associated with medical errors^19^ 4: Stress overload associated with time to start CPR, but not hands on time during resuscitation^20^ 5: Mortality associated with higher rates of burnout, but length of stay not associated with burnout^21^ 6: Emotional Exhaustion associated with standardised mortality ratios, burnout not associated with length of stay^22^
8	Mossburg et al. (2021)	Systematic review 15:4 (Burnout)	Burnout Engagement	Mixed results of the relationship between burnout and errors could be due to a disparate relationship with perceived versus observed errors	1: Burnout was not associated with medical errors^23^ 2: Burnout associated with increased rates of urinary tract infection and surgical site infections^24^ 3: Burnout was not associated with mortality or morbidity^25^ 4: Burnt-out residents made more errors than non-burnt-out residents when using subjective self-reported, but not objective, measures of error^26^

^1^Welp et al. (2015), ^2^Cimiotti et al. (2012), ^3^Tawfik et al. (2018), ^4^Garrouste-Orgeas et al. (2015), ^5^Rabatin et al. (2016), ^6^Saleh et al. (2014), ^7^Linzer et al. (2009), ^8^Dollarhide et al. (2014), ^9^Dugan et al. (1996), ^10^Fahrenkopf et al. (2008), ^11^Garrouste-Orgeas et al. (2015), ^12^Eltaybani et al. (2021), ^13^Schlak et al. (2021), ^14^Mohr et al. (2021), ^15^Vogus et al. (2014), ^16^Hunziker et al. (2012), ^17^Merlani et al. (2011), ^18^Davenport et al. (2007).

The majority of reviews included a small proportion of studies that specifically examined objective measures of patient safety, while one review did not include any studies on objective patient safety outcomes (see Table 1 for details).

Table 1 highlights discrepancies between review-level conclusions and the specific evidence relating to objective patient safety outcomes. To examine these discrepancies in greater detail, we compare and contrast the quantitative relationships reported, followed by a consideration of the higher-order methodological, validity, and scope-related challenges posed by the reviews.

### 2.1 Quantitative associations

Across the eight reviews, a consistent pattern emerges: objective safety outcomes are rarely assessed, and when they are, findings are often inconsistent or null. In the review by Jun et al. (2021), which focused on nurse burnout, higher emotional exhaustion was a significant predictor of increased mortality in one study. In the Garcia et al. (2019) review, only two studies had findings related to objective patient outcomes: burnout was not associated with healthcare-associated infections or medical errors. Dewa et al. (2017), in a systematic review of physician burnout and quality of healthcare, included 12 studies, but only one assessed patient safety objectively and found no relationship between burnout and medical errors. In Hall et al. (2016) systematic review of wellbeing, burnout and patient safety, only seven studies included objective measures, and of these, only two studies assessed burnout. In the first, burnout among residents was not associated with objective measures of error, while depression (but not burnout) was a risk factor for error as measured via chart audit. In the second, residents who scored higher on burnout made more errors when using subjective self-reported, but not objective measures of error.

Li et al. (2024), in a meta-analysis of nurses’ burnout, included 85 studies, but only four assessed objective patient safety outcomes. In one study, emotional exhaustion was associated with rates of pneumonia and pressure ulcers, but not with rates of urinary catheter use or tube feedings in both bivariate and multivariate analyses. Conversely, reduced personal accomplishment was associated only with rates of urinary catheter use in the bivariate analysis and only with rates of tube feeding in the multivariate analysis. In a second study, significant relationships between nurse burnout and failure to rescue and length of stay even after adjusting for patient and hospital characteristics were reported. In the two remaining studies, there was no association between mortality rate and burnout, or adverse events and burnout.

The systematic review of surgeon burnout and patient safety of Al-Ghunaim et al. (2022) included only subjective measures of patient safety, however it was noteworthy that in all nine studies included in the meta-analysis, there were no significant associations between the depersonalisation component of burnout and medical errors. Maslach and Leiter (2016) have argued that the cynicism/depersonalization component is the core element of burnout. Mossburg and Himmelfarb (2021), in their review of burnout and engagement, found mixed results concerning the relationship between burnout and errors, which could be due to a disparate relationship between perceived versus observed errors. Equally, Welp and Manser (2016), in a review of teamwork and burnout, found limited and mixed support for the associations between burnout, teamwork and safety.

Overall, across reviews, the small number of studies assessing objective patient safety outcomes, the predominance of cross-sectional designs, and the heterogeneity of findings substantially limit the strength of inferences that can be drawn. Evidence of associations between burnout and objective safety outcomes appears inconsistent across settings, burnout dimensions, and outcome types, and is often absent altogether. As a result, robust conclusions regarding the role of burnout in explaining objective patient safety outcomes remain difficult to establish.

In the following sections, we examine the higher-order conceptual and interpretive themes that help to explain these patterns and clarify how burnout has been positioned within the patient safety literature.

### 2.2 Higher-order themes

Our CIS approach provided the opportunity to identify higher-order themes to clarify how burnout matters, under what conditions and through which mechanisms. In the following section, we have highlighted four key higher-order themes that help us to elucidate the narratives in the literature that are blocking progress towards a comprehensive understanding of the phenomenon.

#### 2.2.1 Narrow focus on physicians and nurses in healthcare

The vast majority of evidence synthesised in the examined reviews comes from nurses and doctors. This narrow focus overlooks large segments of the healthcare workforce, such as allied professionals, ancillary, administration, reception, and catering staff. These groups constitute a significant portion of the workforce, yet their views are consistently underrepresented. Much of this “hidden workforce” has direct patient contact, and is essential to health and social care organisations; thus their impact on patient outcomes should be as much a cause for concern as are clinical staff experiences. This limitation is particularly pronounced in long-term care and community-based services, where the delivery of care relies heavily on healthcare assistants, support workers, and other ancillary staff who provide substantial direct patient contact. In many such settings, these staff constitute the majority of the workforce and are central to day-to-day care processes, continuity, and monitoring (Buchan et al., 2019; World Health Organization, 2020; OECD, 2020). Their relative absence from the burnout–patient safety literature therefore represents not only an occupational gap but a structural blind spot in understanding how safety is enacted across the wider health and social care system. Further, this “hidden workforce” disproportionately comprises lower-paid workers, women, and staff from minority ethnic backgrounds, with a higher representation of minority ethnic groups in non-clinical roles. Thus, the exclusion of these groups therefore risks reproducing structural blind spots in both safety and wellbeing research.

By concentrating primarily on nurses and physicians, the literature also reinforces an individualised view of patient safety that sidelines the team-based and collective nature of healthcare work, implicitly suggesting that safety can be improved by addressing burnout symptoms at the individual level. Researching at the team or unit level rather than individuals may provide a more meaningful insight into the relationship between patient safety outcomes and burnout. Aggregated measures of burnout capture shared exposure to working conditions, such as sustained workload, staffing adequacy, and resource constraints, rather than transient individual states. Objective safety outcomes, such as mortality, healthcare-associated infections, and failure to rescue, are produced through collective processes including coordination, communication, and the adaptive capacity of teams at the unit level. As a result, analyses that aggregate burnout to the unit or hospital level are better aligned with the mechanisms through which these outcomes are produced. For example, Welp et al. (2015) aggregated clinicians’ emotional exhaustion to the ICU level and found that units with higher mean exhaustion had higher standardised mortality ratios. Similarly, Cimiotti et al. (2012) aggregated nurse burnout to the unit or hospital level and reported associations with healthcare-associated infections. In both cases, burnout was treated not as an individual risk factor for error, but as a collective indicator of working conditions that shape the capacity of units to deliver safe care.

#### 2.2.2 Lack of theoretical positioning of burnout in the patient safety monitoring system

All reviews report on the lack of theoretical background in their reviewed studies. Table 2 presents details on the theoretical frameworks across the reviews. At the review level, most reviews did not adopt an overarching theoretical framework but instead synthesized empirical findings and made recommendations for practice, policy and/or measurement. The limited engagement with theory could be partly related to journal formats (especially in medical journals), which often prioritise concrete implications over theoretical discussion. Theories were mentioned in many of the papers, but these references often aligned with what has been called “theory salting” (Spector, 2024), a particular problem in the organizational science where articles are ‘salted’ with theories without substantive integration or explanatory frameworks.

**Table 2:** Review of theoretical frameworks.

Review no.	Study	Theory or framework used to support safety outcomes	How psychological variables are incorporated within safety outcomes
1	Jun et al. (2021)	The Quality Health Outcome (QHO) model guided the review for its incorporation of the complex and multi-directional relationships among the three elements of the traditional structure-process-outcome model (Mitchell et al., 1998). According to the QHO model, the relationships among the system, intervention, client, and outcomes are dynamic and reciprocal, thus analysis of each component is necessary to provide a comprehensive picture of the complexity of patient care in healthcare settings.	Safety improvement should focus on upstream organisational determinants that shape psychological strain and thereby influence care processes and outcomes.
2	Garcia et al. (2019)	No specific theory considered, relevant literature covered and recommendations on practice/policy and measurement.	The paper positions safety as vulnerable to ineffective teamwork, failed organisational processes, and psychological overload of workers.
3	Dewa et al. (2017)	They incorporate the framework from WHO and the IOM who suggest six dimensions for quality of healthcare: effectiveness, efficiency, accessibility, equitability, acceptability and safety. This review focuses on two dimensions of quality: acceptability (i.e., patient satisfaction, perceived quality of care and communication) and safety (i.e., minimising risks or harm to patients). Two dimensions were chosen because they reflect the quality of patient-physician interactions.	Frames safety as one of the quality dimensions, and argues burnout can degrade the quality of patient–physician interactions, linking psychological strain to both acceptability (communication, satisfaction) and safety (minimising risk and harm). Causal direction remains uncertain in many included studies, as safety measures can mix objective events with reporting behaviour.
4	Hall et al. (2016)	Reviewed literature concerning human factors, wellbeing and burnout. No specific theoretical model presented.	Safety was treated as a function of organisational conditions shaping staff wellbeing, with psychological mechanisms operating through fatigue, distress, and reduced cognitive and relational capacity, which increase error risk. Poor wellbeing, as characterized by depression, anxiety, poor quality of life and stress, and high levels of burnout, were found to be significantly associated with more self-reported errors, with a smaller number of studies showing an association of these factors with objective measures of error.
5	Li et al. (2024)	No specific theory considered, relevant literature covered and recommendations on practice/policy and measurement.	Noted that anti-burnout efforts have focused on individual interventions. In terms of patient safety outcomes, studying processes at the work unit level, where health care workers experience teamwork, feelings of community, professional development, and recognition, is recommended.
6	Al-Ghunaim et al. (2022)	No specific theory considered, relevant literature covered and recommendations on practice/policy and measurement.	Stronger causal claims were limited by predominance of observational designs in the primary evidence base, and by heterogeneity in how “patient safety” was measured across included studies.
7	Welp and Manser (2016)	Review drew from the theoretical foundations of the reviewed studies and from psychological theories relevant to the topic to aid interpretation of the findings and formulate hypotheses. For example, using the job demands-resources model, they propose that teamwork can be a demand or a resource. The model proposes two parallel processes that influence positive and negative aspects of occupational well-being, such as work engagement and burnout.	Review suggested that teamwork processes influence safety directly, and also shape clinician wellbeing, which in turn affects performance and safety, aligning psychological strain with system performance pathways.
8	Mossburg and Himmelfarb (2021)	No detailed theory was described. They shortly refer to job demands-resources model. Authors drew upon the view that job demands contribute to burnout while job resources promote engagement.	Review suggested burnout and engagement as individual-level psychological variables that can level up to unit-level and safety culture. Additionally, they frame safety culture as a group-level phenomenon shaped by observable behaviours.

The more substantive problem, however, is not simply the superficial use of theory, but the lack of explicit recognition that this absence limits what can be inferred from the evidence. Theory testing requires clear specification of constructs, mechanisms, and expected relationships; without this, empirical findings remain difficult to interpret or integrate. As a consequence, burnout is rarely positioned with robust theoretical justification within patient safety systems. Across studies, burnout is variously implied to be an antecedent, mediator, correlate, or sometimes outcome of patient safety processes, but its temporal ordering, level of operation (individual, team, or organisational), and/or causal pathways are typically left unspecified. This lack of theoretical positioning is not entirely surprising given the methodological characteristics of the literature. The majority of studies synthesised in the reviewed papers employing cross-sectional designs, limiting the ability to specify temporal ordering or to distinguish between burnout as a potential antecedent, correlate, or consequence of patient safety outcomes. Importantly, many of the primary studies included in the eight reviews did not systematically measure factors that are theoretically central to patient safety but lie between burnout and clinical outcomes. Variables such as the quality of clinical handovers, the level of supervision of doctors in training, and the continuity of care provided by the multidisciplinary team were rarely assessed, despite their well-established relevance for safety. This reinforces the tendency to treat burnout as a proximal/direct explanatory factor, rather than as part of a broader system of conditions and processes that shape patient safety outcomes.

#### 2.2.3 Exaggeration of conclusions beyond objective/reported implications of causality

As previously mentioned, all reviews noted the predominance of cross-sectional data, which limits attempts to infer causality or direction of relationships. Despite this shared methodological limitation, there was a discernible difference how the findings were narratively interpreted. On the one hand, the evidence concerning the relationship between burnout and self-reported safety culture was generally considered good evidence. The limitations were noted, but review authors often accepted the evidence as a good basis to inform practice and/or policy – as was the case for burnout and its significant relationships to objective patient safety outcomes. In contrast, null or contradictory findings concerning the relationship between burnout and objective patient safety outcomes were subject to extensive *post hoc* explanation and were rarely allowed to challenge the broader narrative that burnout plays a crucial role in patient safety. The considerable heterogeneity in how both burnout and patient safety outcomes were measured, further complicates attempts to integrate findings across studies. As a result, less than half of the reviews (3 out of 8) were able to conduct a meta-analysis, with heterogeneity and lack of comparability cited as primary barriers. Where meta-analyses were conducted, the small number of included studies, aside from Li et al. (2024), further constrained the ability to examine moderators or to assess the robustness of observed associations.

This pattern reflects an exaggeration or asymmetry of warranted conclusions, whereby supportive evidence is taken at face value while non-supportive evidence is explained away. From the perspective of warranted assertability (Dewey, 1939), claims about the role of burnout in patient safety should be proportionate to the strength, consistency, and scope of the available evidence, and remain open to revision in light of contradictory findings. Combined with the lack of consistent theoretical positioning and justification identified earlier, the inconsistent treatment of contradictory findings represents a missed opportunity to generate alternative hypotheses about the role of burnout, including its potential position as a consequence of safety failures, a contextual indicator of system strain, or a marker of broader organisational dysfunction. Without engaging seriously with these possibilities, the literature risks reinforcing causal narratives that exceed what can be currently warranted by the evidence.

#### 2.2.4 Objective events or reported events?

The present paper focused on objective patient safety outcomes rather than subjective measures of patient safety. As discussed earlier, objective patient safety outcomes are not inherently superior to subjective patient safety assessments. For example, measures based on incident reporting can be unreliable, with many epidemiological problems related to underreporting (Noble and Pronovost, 2010), while incident reporting can be more robust when near-misses and “good catches” are also measured.

From the perspective of warranted assertability (Dewey, 1939), claims based on objective safety outcomes require careful consideration of the conditions under which these outcomes are generated, detected, classified, and recorded. The central issue is therefore not simply whether events occurred, but whether the inferences drawn from recorded events are justified given the social, organisational, and measurement processes that shape their visibility. The relationship between psychological safety and objective patient safety outcomes is an illustrative parallel. Quantitative evidence is equivocal, and the same indicator, reporting of safety incidents, has been associated in some studies with higher levels and in other studies with lower levels of psychological safety (Montgomery et al., 2025). Without explicit attention to contextual contingencies, objective indicators are at risk of being treated as straightforward facts when, in reality, they embody complex behavioural and organisational processes. This interpretive ambiguity also applies to burnout. While burnout is often assumed to reduce attention, engagement, or vigilance, increased reporting alone does not mean increased occurrence of avoidable safety incidents. In fact, emotionally exhausted staff operating in chronically unsafe environments may become hypervigilant or change patient-safety related behaviours as a coping or protective response (e.g., Khammissa et al., 2022, who found higher burnout associated with more referrals and ordering of more tests in physicians). In such cases, higher levels of recorded safety incidents may reflect increased detection or reporting (Garcia et al., 2019) rather than deteriorating safety performance. Thus, when these processes are not measured, controlled for, or theoretically integrated, conclusions about the relationship between burnout and patient safety risk exceeding what the available evidence can warrant.

## 3 General discussion

Our paper builds upon the 2004 review by Hoff et al. (2004), which examined the potential role of organizational factors in enhanced patient safety and medical error prevention. In line with this review, our findings indicate that there is little evidence for asserting the direct influence of job burnout on patient safety outcomes. Our critical interpretative synthesis extends the literature in the following ways.

Firstly, the evidence concerning burnout is mixed and inconclusive, raising some uncertainty. As highlighted in Table 1, there is a dissonance in that burnout appears to be related to patient safety at a generic or perceptual level, but a more detailed assessment of the evidence indicates that there are more questions than answers about this relationship. Second, the evidence is drawn from a narrow range of experiences, methodologies and theoretical perspectives. The predominant focus on the experiences of frontline staff (nurses and physicians) in acute settings significantly limits our understanding. Additionally, the individuals responding to survey research may represent a further bias towards healthier employees (the healthy worker effect). Overall, we run the risk of building our evidence base on a narrow range of experiences that ignores the social and economic hardships that impact the wider pool of individuals working in health and social care. Considerable evidence has established that rates of morbidity and mortality are arrayed by socioeconomic status (SES), race/ethnicity, or stigmatised identity (Phelan and Link, 2013). Notably, such health inequalities were absent from the reviews included in this study. As a result, existing theories and empirical evidence about patient safety outcomes insufficiently account for how employee SES shapes the wellbeing and safety related behaviours in demanding healthcare environments. Third, the evidence and its limitations constrain the development of effective interventions targeting burnout or patient safety, as what is available lacks the required specificity in terms of delineating mechanisms, directions of effects and system-level confounders. Without clear specification of where burnout operates in the system, through which mechanisms it influences safety, and under what conditions these processes unfold, intervention efforts risk targeting symptoms rather than system-level causes. Extrapolating from momentary snapshots to large scale change is problematic.

More generally, there is a need to locate burnout within a coherent theoretical framework that specifies how different burnout dimensions operate within healthcare systems Recent longitudinal evidence indicating that burnout development fits more closely with a strain rather than a stressor theory (Maunz et al., 2026), is particularly relevant for healthcare, where burnout feelings can be treated as a norm and an acceptable byproduct of ‘good’ performance (Montgomery et al., 2019). Given its ubiquity across health and social care and its role as an indicator of organizational strain, burnout may be an important contextual factor in understanding how employee interactions contribute to patient safety outcomes. However, its role as a potential confounder rather than a direct driver needs to be better understood. For example, Fahrenkopf et al. (2008) observed a discrepancy between the results of chart audits and physician self-report; those with higher burnout scores reported higher numbers of medical errors than the chart audits would suggest. The evidence demonstrating that burnout can be contagious in healthcare settings (Bakker et al., 2005) is particularly relevant here, as such contagion has the potential to “bleed into” related organizational variables such as leadership style, team atmosphere, and interpersonal dynamics in working teams. Demerouti and Bakker (2025) argue that to revitalise the field and drive meaningful progress, burnout research must evolve beyond mere quantification and adopt stronger theoretical frameworks and innovative methodologies that can unpack the complex mechanisms underlying the burnout syndrome.

Additionally, it would be helpful for research to assess parallel variables, such as work engagement, and to compare and contrast different patterns (e.g., high burnout only, highly engaged only, and both highly engaged and burned out). Highly committed vocational employees, such as health workers, are ones who can experience engagement and burnout developing in tandem. Profiling the different types of burnout and engagement (as recommended by Leiter and Maslach, 2016) has the potential to elucidate better the mechanisms of patient safety. For example, especially within healthcare, it would be useful to distinguish between different engagement modes, such as personal role engagement, engagement as management practice and self-engagement with performance (Janes et al., 2021).

### 3.1 Implications for research

In terms of connecting burnout and patient safety outcomes, the evidence reviewed here suggests that patient safety is more plausibly understood as an emergent property of dynamic socio-technical systems, arising from interactions between psychological processes, organisational structures, technologies, and latent system conditions. From an epistemological perspective, this calls for greater caution in research design, particularly with respect to drawing (incorrect) causal inferences from proxy measures and cross-sectional associations that do not test mechanisms, temporal dynamics, and contextual contingencies. Many studies rely on linear statistical models that assume stable, additive relationships between burnout and safety outcomes. While such models are often appropriate and informative, the presence of a statistically significant linear association at the between-person level does not imply that the underlying safety processes are themselves linear, uniform, or context-independent. In complex socio-technical systems, safety-relevant effects may be contingent, threshold-based, or episodic, becoming visible only under particular conditions. Greater attention to alternative ways of structuring data and modelling relationships will allow for better mapping of whether or how psychological/organisational determinants contributed to their occurrence, detection, or mitigation.

The limited success that has been achieved in developing evidence to link burnout and patient safety means that bottom-up approaches are needed. Co-design and co-produced approaches represent an effort to more meaningfully engage stakeholders in designing research aiming to understand and address healthcare problems (Findlay et al., 2024; Redman et al., 2021). Such approaches are more likely to ensure that the theory, research questions, and methodology are better aligned with and reflective of the complex realities of healthcare and patient safety (Koskela-Huotari et al., 2013). Co-design approaches have recently been used to address burnout and moral distress among health workers in long-term care sector (Boamah et al., 2025). Congruently, co-design approaches have used to develop “restorative learning” approaches to patient safety among patients and healthcare professionals (O’Hara et al., 2025). The challenge going forward is to ensure that co-design approaches avoid the mistakes of the past (e.g., narrow range of experiences) and can be integrated into more holistic theories about job burnout and patient safety.

### 3.2 Implications for theory: do we need a new open systems approach to burnout in healthcare?

In the absence of an explicit systems-oriented framework, burnout is variously treated as an antecedent, correlate, or outcome of patient safety without clear justification, leading to reliance on cross-sectional designs, individual-level surveys, and underspecified models. This conceptual ambiguity is reflected in methodological choices that also reflect deeper theoretical uncertainty about where burnout sits, at what level it operates, and through which mechanisms it might matter for safety outcomes. An open systems approach provides a way to resolve this impasse by situating burnout within the organisational conditions under which care is delivered. Rather than positioning burnout as a direct causal predictor, an open systems perspective conceptualises burnout as an indicator of both organizational dysfunction and wider systemic forces, including economic instability, political regulation, cultural expectations, and ecological stressors, that influence how individuals navigate the competing demands of performance and wellbeing. For example, related work in Information Systems and organisational theory has examined how technological infrastructures, accountability systems, and organisational routines structure clinicians’ work and experiences of strain. Socio-technical models such as the Systems Engineering Initiative for Patient Safety (SEIPS) model (Carayon et al., 2013) and the organisational routines theory (Pentland and Feldman, 2005) conceptualise healthcare performance as emerging from dynamic interactions between technologies, formal processes, tasks, environments, and human actors. However, despite their theoretical sophistication, these approaches have not consistently translated into a cumulative quantitative evidence base demonstrating clear and reproducible associations between burnout and objectively recorded patient safety outcomes in the review and meta-analytic literature synthesised to date. Moreover, much of this work remains primarily intra-organisational in scope and heavily focused on technological or workflow configurations. Our proposed open-systems perspective extends beyond this boundary by explicitly situating burnout within broader socio-economic, regulatory, political, and labour-market conditions that shape healthcare systems from outside as well as within. The aim is therefore not to replace existing socio-technical models, but to argue that burnout cannot be adequately theorised or empirically evaluated without accounting for these wider structural forces. While the importance of moving beyond individual behaviour in burnout research has been noted previously (e.g., Goroll, 2020; Montgomery et al., 2019), the interdependence of burnout with the social milieu and historical context has not been fully exploited. This is a gap in the research.

The socio-technical approach has the potential to more successfully capture the contextual shape of both burnout and patient safety practises. For example, underserved areas and highly deprived areas in healthcare face significant challenges in maintaining a sustainable healthcare workforce (Park et al., 2024; Wise, 2023), and in delivering care, complicated by other factors such as high patient demand, limited resources, and lower levels of staff retention (Wise, 2023). The narrow occupational focus of existing evidence means that socioeconomic conditions shaping burnout and safety are insufficiently incorporated into prevailing models. Arguing for an open systems approach to burnout does not negate the importance of the internal processes. For example, the assumptions of the JD-R model with regard to the relationship between job demands and job resources have been largely validated by research (Lesener et al., 2019; Guthier et al., 2020). An open-systems perspective also invites consideration of related constructs such as the second victim phenomenon, moral distress, and moral injury, which reflect different forms of staff psychological strain in response to adverse events and moral pressures. However, their direct relationship with objectively recorded patient safety outcomes remains under-examined, representing an important area for future research.

The open systems approach provides the opportunity to delineate the structural and societal elements that drive internal processes in organisations. The benefits of a socio-technical approach to burnout include; recognising the reality that as globalization increases wellbeing is pushed and pulled by forces seemingly distal to the influence of specific organizations and industries, better representing the messy nature of organizations, widening the methodological and theoretical horizons of research on burnout, bringing a historical and contextual analysis to burnout, and integrating the influence of professional bodies that are both inside and outside the organization (e.g., professional associations) (Montgomery, 2025). Our models of burnout in health and social care need to reflect the reality that the majority of employees are underpaid, have low career mobility, can perform uninteresting/repetitive work, have limited job autonomy and devote considerable energy to economic survival rather than continuous professional development.

### 3.3 Implications for practice

The findings of this synthesis have important implications for evidence-based practice in patient safety. While evidence-based approaches are widely promoted, our review highlights that evidence alone does not guarantee good practice. From a warranted-assertability perspective, evidence should inform practice only to the extent that the claims derived from it are proportionate to its consistency, and interpretive clarity; this means that where evidence is cross-sectional, heterogeneous, or theoretically under-specified, strong prescriptive recommendations risk exceeding what can really be supported. Implementing interventions on the basis of largely mixed and confusing evidence risks misdirecting resources, placing responsibility on individuals for system-level problems, and overlooking organisational conditions that shape both burnout and safety. Rather than asking *what intervention does the evidence support*, practitioners and policymakers should ask *what claims does the evidence actually warrant*. Aligning practice with warranted assertability does not weaken evidence-based practice, but it strengthens it by ensuring that interventions are proportionate with the quality and consistency of the available evidence.

### 3.4 Limitations

Although the reviewed studies report objective patient safety outcomes (e.g., complications, adverse event rates etc.), they do not make explicit whether these outcomes were preventable, nor do they systematically evaluate whether or how job burnout contributed to their occurrence, detection, or mitigation. This limits interpretability and analyses of the data and their role within policy utility. Without structured classification of harms by preventability and contributory mechanisms, outcome measures risk collapsing fundamentally different phenomena into an incorrect category or direction. Adverse events may arise from unavoidable risk given clinical complexity, yet even in such cases there may be some preventable components worth examining, such as situational awareness, failures in communication, event escalation, or post-event learning. Safety science requires linked advances and safety measurements that are meaningfully understood and not just reported to bridge the gaps between the real-world challenges of clinical work and objective patient safety outcomes.

A limitation of the present study is that we did not quantify or formally assess the degree of overlap in primary studies across the included systematic reviews. Overlap in primary studies is an expected feature of syntheses examining a mature literature, and in the context of a critical interpretive synthesis such overlap is analytically less important than how evidence is taken up, framed, and extended at the review level. Our interests lay in how similar bodies of evidence were used to support different—or sometimes increasingly strong—claims about burnout and patient safety, rather than in quantifying the extent to which reviews relied on the same studies. From this perspective, overlap was viewed as part of the discursive and evidential context within which claims were produced, rather than as a source of bias that required adjustment.

A Critical Interpretive Synthesis approach offers an advantage over the traditional systematic review approach, in that it allows us to reframe and reinterpret existing literature through a synthesising argument that could generate new insights and highlight research gaps (Dixon-Woods et al., 2006). However, such an approach involves various limitations. Firstly, the synthesis reflects our team’s interpretation, shaped by the focus and theories of the included papers. Secondly, given the range and number of topics covered by patient safety, it is possible that we may have missed relevant reviews. Given that our goal was conceptual and empirical saturation rather than finding every paper on a topic, such a pragmatic trade-off was acceptable. Thirdly, we were forced to take an overly inclusive approach to labelling variables as patient safety outcomes. However, a detailed reading of the reviewed papers revealed that some of the included variables were proxy measures of patient safety (e.g., non-routine event and/or unusual event). Going forward, we need a clearer taxonomy of what constitutes objective patient safety outcomes.

## 4 Conclusion

The field of job burnout has matured over the last 40 years, and there is an acceptance that it should play a significant role in both medical error and patient harm. However, the available evidence linking burnout to objective patient safety outcomes remains equivocal. More importantly, our synthesis suggests that advancing this field requires a socio-technical approach to burnout that reflects the realities of healthcare work as shaped by social, economic, and organisational constraints, rather than treating burnout as an isolated individual-level risk factor. Where associations are observed, they are highly contingent on how both burnout and safety are conceptualised, measured, and interpreted, and are often absent when outcomes are assessed independently of self-report.

Objective patient safety outcomes are frequently treated as definitive indicators of harm, yet they are co-produced through behavioural, organisational, and measurement processes that are rarely acknowledged or modelled. As a result, associations between burnout and recorded safety events may reflect differences in detection, reporting, adaptation, or system functioning, rather than straightforward deterioration in care.

Taken together, the evidence reviewed here challenges the assumption that burnout can be meaningfully understood or effectively addressed as an individual-level risk factor for patient safety. Instead, burnout is better conceptualised as a system-level indicator of organisational strain, embedded within broader socio-technical, economic, and regulatory contexts that shape both staff wellbeing and the conditions for safe care. An open-systems perspective allows burnout to be situated alongside other interacting determinants of safety, without requiring it to function as a direct causal driver of harm.
